# The relationship between YKL-40 and preeclampsia remains controversial

**DOI:** 10.1590/1806-9282.20241165

**Published:** 2024-12-02

**Authors:** Lianlian Song, Huaxiao Tang

**Affiliations:** 1 Qingdao University, Weihai Second Affiliated Hospital (Weihai Maternal and Child Health Hospital), Department of Pathology – Weihai, China.

Dear Editor,

We were very pleased to read the study by Queiroz^1^, and his colleagues in which they revealed that the rs880633 polymorphism may serve a protective role against the development of preeclampsia, whereas the rs7515776 polymorphism may be associated with an elevated risk. This study is a promising direction for future research, suggesting that the rs880633 polymorphism in the CHI3L1 gene could act as a protective factor against preeclampsia. However, some views should be raised in my view.

The first major issue is that the study should first examine whether YKL-40 is elevated in patients with preeclampsia. The Chitinase 3-Like 1 (CHI3L1) gene encodes for a glycoprotein, also known as YKL-40. Because the link between YKL-40 and preeclampsia remains controversial, previous studies found that YKL-40 concentrations in cord serum were significantly higher in pre-eclamptic pregnancies^2^. Then another study found no link between YKL-40 and preeclampsia^3^. Therefore, clarifying the correlation between YKL-40 and preeclampsia is an important research basis.

Second, it is necessary to clarify whether there is a link between CHI3L1 gene polymorphisms and YKL-40. In other words, whether CHI3L1 gene polymorphism affects serum YKL-40 in patients with preeclampsia. If CHI3L1 polymorphisms do not affect serum YKL-40 levels, there is no point in studying the association between CHI3L1 polymorphisms and preeclampsia.

In summary, the authors should first clarify whether there is a relationship between YKL-40 and preeclampsia, then clarify whether the CHI3L1 gene polymorphism affects serum YKL-40 in patients with preeclampsia, and finally determine the relationship between the CHI3L1 gene polymorphism and preeclampsia.
